# Age-Stratified Seroprevalence of Cytomegalovirus and Epstein–Barr Virus in a Population-Based Cohort in Japan, 2017–2024

**DOI:** 10.1093/ofid/ofag678

**Published:** 2026-11-04

**Authors:** Mami Ishikuro, John D Diaz-Decaro, Kenichi Noguchi, Taku Obara, Masatsugu Orui, Kazuki Kumada, Hisaaki Kudo, Soichi Ogishima, Naoki Nakaya, Atsushi Hozawa, Shinichi Kuriyama, Kumi Kawaji, Steffi Naomi, Susan A Hall, Laura H Hendrix, Katja Engelberg, Anita Iyer, Enejda Senko, Osamu Kogawara, Masayuki Yamamoto, Eiichi N Kodama

**Affiliations:** Tohoku Medical Megabank Organization, Tohoku University, Sendai, Japan; Graduate School of Medicine, Tohoku University, Sendai, Japan; Development, ID/Rare/Therapeutics, Moderna, Inc., Cambridge, Massachusetts, USA; Tohoku Medical Megabank Organization, Tohoku University, Sendai, Japan; Tohoku Medical Megabank Organization, Tohoku University, Sendai, Japan; Graduate School of Medicine, Tohoku University, Sendai, Japan; Tohoku Medical Megabank Organization, Tohoku University, Sendai, Japan; Graduate School of Medicine, Tohoku University, Sendai, Japan; International Research Institute of Disaster Science, Tohoku University, Sendai, Japan; Tohoku Medical Megabank Organization, Tohoku University, Sendai, Japan; Advanced Research Center for Innovations in Next-Generation Medicine, Tohoku University, Sendai, Japan; Tohoku Medical Megabank Organization, Tohoku University, Sendai, Japan; Tohoku Medical Megabank Organization, Tohoku University, Sendai, Japan; Advanced Research Center for Innovations in Next-Generation Medicine, Tohoku University, Sendai, Japan; Tohoku Medical Megabank Organization, Tohoku University, Sendai, Japan; Tohoku Medical Megabank Organization, Tohoku University, Sendai, Japan; Graduate School of Medicine, Tohoku University, Sendai, Japan; Tohoku Medical Megabank Organization, Tohoku University, Sendai, Japan; Graduate School of Medicine, Tohoku University, Sendai, Japan; International Research Institute of Disaster Science, Tohoku University, Sendai, Japan; Tohoku Medical Megabank Organization, Tohoku University, Sendai, Japan; Graduate School of Medicine, Tohoku University, Sendai, Japan; International Research Institute of Disaster Science, Tohoku University, Sendai, Japan; Development, ID/Rare/Therapeutics, Moderna, Inc., Cambridge, Massachusetts, USA; Development, ID/Rare/Therapeutics, Moderna, Inc., Cambridge, Massachusetts, USA; Development, ID/Rare/Therapeutics, Moderna, Inc., Cambridge, Massachusetts, USA; Development, ID/Rare/Therapeutics, Moderna, Inc., Cambridge, Massachusetts, USA; Development, ID/Rare/Therapeutics, Moderna, Inc., Cambridge, Massachusetts, USA; Clinical Development, ID/Rare/Therapeutics, Moderna Japan Co., Ltd., Tokyo, Japan; Tohoku Medical Megabank Organization, Tohoku University, Sendai, Japan; Graduate School of Medicine, Tohoku University, Sendai, Japan; Tohoku Medical Megabank Organization, Tohoku University, Sendai, Japan; Graduate School of Medicine, Tohoku University, Sendai, Japan; International Research Institute of Disaster Science, Tohoku University, Sendai, Japan

**Keywords:** age-specific prevalence, cytomegalovirus, Epstein–Barr virus, seroepidemiology, Japan

## Abstract

**Background:**

Cytomegalovirus (CMV) and Epstein–Barr virus (EBV) are ubiquitous latent herpes viruses, but contemporary population-based seroprevalence data in Japan remain limited, particularly in younger age groups. This study aimed to characterize age- and sex-specific CMV and EBV seroprevalence in a population-based Japanese cohort and to describe combined serostatus patterns across age groups.

**Methods:**

We conducted a cross-sectional seroepidemiological analysis of the Tohoku Medical Megabank Project Birth and Three-Generation (TMM BirThree) cohort. Serum samples collected between 2017 and 2024 were tested for CMV-IgG (age 4–40 years; n = 2247) and EBV viral capsid antigen IgG (age 4–30 years; n = 1647) using commercial ELISA. Seroprevalence was stratified by age and sex. Combined CMV/EBV serostatus patterns were described, and sensitivity analyses assessed temporal consistency and intra-family clustering.

**Results:**

Cytomegalovirus seroprevalence increased during childhood and adolescence and plateaued at approximately 70%–80% in early adulthood, whereas EBV seroprevalence rose steadily, exceeding 70% in late adolescence and 90% in the late 20s, with minimal sex differences. Dual seropositivity increased with age, the CMV−/EBV− group declined, and CMV−/EBV+ remained more common than CMV+/EBV− across all age groups. These patterns were consistent in sensitivity analyses.

**Conclusions:**

Cytomegalovirus and EBV exhibit distinct age-related seroepidemiologic patterns in Japan despite overlapping transmission routes. The early plateau of CMV seroprevalence and near-universal EBV seropositivity by young adulthood indicate divergent infection dynamics with implications for congenital CMV prevention, infectious mononucleosis risk, and ongoing vaccine strategy.

## Teaser

In this population-based Japanese cohort, CMV seroprevalence plateaued by adulthood, whereas EBV approached near-universal seropositivity. These distinct age-related patterns provide epidemiologic evidence relevant to public health planning and to evaluation of future preventive strategies, including vaccination and screening approaches.

Human cytomegalovirus (CMV) and Epstein–Barr virus (EBV) are ubiquitous herpesviruses that establish lifelong latent infections and are primarily transmitted through close contact with saliva and bodily fluids. Cytomegalovirus infection is a major public health concern because primary infection during pregnancy can result in miscarriage, stillbirth, and congenital abnormalities and is a major cause of nongenetic congenital hearing loss and neurodevelopmental impairment worldwide [1]. Cytomegalovirus infection also poses clinical challenges in immunocompromised settings, including transplantation and intensive immunomodulatory therapy [2]. Epstein–Barr virus is implicated in infectious mononucleosis and multiple chronic conditions, including Burkitt's lymphoma [3, 4], Hodgkin's lymphoma [5, 6], gastric cancer [7], nasopharyngeal cancer [8], and autoimmune conditions, most notably multiple sclerosis [9]. Although CMV and EBV are of considerable public health importance, their epidemiology remains incompletely characterized across many populations.

In Japan specifically, data on CMV and EBV seroprevalence, especially among healthy children and young adults, remain limited. Cytomegalovirus seroepidemiology has been studied primarily in pregnant women [10–12], transplant recipients [13], and elderly blood donors [14]. Although CMV seroprevalence shows relatively limited variation across ethnic populations, EBV demonstrates marked racial and geographic heterogeneity. This underscores the need for population-based seroepidemiological data from Japan. Recent changes in hygiene practices, including improved sanitation, as well as shifts in family structure, such as smaller households and declining birth rates, may also have altered transmission dynamics. Yet robust data in healthy children and young adults are lacking. Epstein–Barr virus seroprevalence estimates are largely based on studies conducted more than a decade ago or in special populations [15, 16].

Understanding the current seroepidemiology of CMV and EBV in Japan is important for public health planning. Declining seroprevalence of CMV among women of reproductive age increases susceptibility to primary infection during pregnancy and the risk of congenital CMV [11], a major cause of hearing loss and neurodevelopmental impairment. For EBV, shifts toward later primary infection are linked to higher rates of symptomatic infectious mononucleosis [17] and may provide context for the rising incidence of multiple sclerosis observed in Japan. Both viruses [18, 19] are targets for ongoing vaccine development efforts, and up-to-date population-based seroprevalence data may help inform future vaccine development and evaluation.

This study used the Tohoku Medical Megabank Project Birth and Three-Generation (TMM BirThree) Cohort Study [20] to generate current population-based estimates of CMV and EBV seroprevalence in Japan. Specifically, we characterized the age- and sex-stratified seroprevalence of CMV and EBV-IgG antibodies in children, adolescents, and young adults and explored the patterns of co-infection. These findings clarify the epidemiology of latent viral infections in Japan and support future vaccine development and public health planning, including screening programs.

## MATERIALS AND METHODS

### Study Population

This cross-sectional analysis was conducted within the TMM BirThree Cohort Study, a large family-structured cohort established to investigate the genetic and environmental determinants of health in Japan. Participants, including siblings, parents, and grandparents, were enrolled between 2013 and 2017. Biospecimens (blood samples) were collected and stored at −80°C. The seroprevalence analysis included randomly selected samples collected between July 2017 and April 2024 from individuals aged 4–40 years for CMV and 4–30 years for EBV. If the number of participants in a 3-year band was insufficient, all available biospecimens were used. Age ranges were defined to ensure representation across childhood, adolescence, and young adulthood and to align with sequential assay batches. Participants were selected from among those who had completed detailed secondary or tertiary health surveys and had sufficient stored blood samples. Demographic information, including sex and age at sample collection, was obtained from the centralized database of the Tohoku Medical Megabank Organization (ToMMo).

### Laboratory Testing

Serological testing for CMV and EBV antibodies was performed using commercial enzyme-linked immunosorbent assay (ELISA) kits (Denka Co., Ltd., Tokyo, Japan) according to the manufacturer's instructions. CMV-IgG and EBV-IgG (Viral Capsid Antigen; VCA) levels were quantified to determine lifetime exposure. Positive and negative controls were included in each assay plate. Serostatus (positive, borderline, or negative) for CMV and EBV-IgG was determined according to the manufacturer's instructions.

For quality control, assay reproducibility was evaluated using independent experiments performed in triplicate. Based on this validation, subsequent measurements were performed using a single determination per sample. The potential impact of repeated freeze–thaw cycles was evaluated in a subset of samples, and no pooling of specimens was performed. All assays were performed in the ToMMo Biobank laboratory under appropriate biosafety conditions by a certified clinical laboratory technician.

### Additional EBV Serological Assessment

To further examine whether EBV infection could have been missed by testing VCA-IgG alone, additional serological testing was performed in 88 samples from a purposively selected assay plate. This plate was selected because it contained relatively balanced numbers of VCA-IgG-positive, borderline, and negative samples, thereby providing a sufficient number of borderline and negative samples for evaluating possible serological misclassification. The samples were additionally tested for VCA-IgM, EA-IgG, and EBNA-IgG using commercially available ELISA kits (Denka Co., Ltd.) according to the manufacturer's instructions.

### Statistical Analyses

Seroprevalence was estimated as the proportion of participants who tested positive for CMV-IgG or EBV-IgG antibodies. Estimates were stratified by age (3-year increments) and sex, with corresponding 95% confidence intervals (CIs) calculated using the Wilson score method [21]. Borderline (equivocal) results were uncommon for CMV (maximum 2.7%) but more frequent for EBV (18% in males, 13% in females). For statistical analyses, borderline/equivocal samples were classified as seronegative (see Supplementary Tables 1 and 2).

To assess co-infection patterns, individuals were categorized into 4 groups: CMV+/EBV+, CMV+/EBV−, CMV−/EBV+, and CMV−/EBV−. Age-dependent trends were descriptively assessed and displayed using bar charts.

As participants were recruited from family-structured cohorts, including siblings, parents, and grandparents, a sensitivity analysis was performed to assess the potential impact of intra-family clustering. This analysis restricted the dataset to one randomly selected participant per family member. Age- and sex-stratified CMV and EBV seroprevalence estimates from this restricted dataset were compared with those from the full cohort to evaluate whether the overall patterns were preserved.

Statistical analyses were performed using R version 4.3.0 (R Foundation for Statistical Computing, Vienna, Austria).

## RESULTS

### Study Population

Overall, 2247 participants aged 4–40 years were included in the CMV seroprevalence analysis and 1647 participants aged 4–30 years in the EBV analysis (Table 1). As shown in Table 1, participants were randomly selected by sex, with 100 males and 100 females in each 3-year age stratum. The age groups were designed to broadly represent educational stages rather than school-year alignment: 4–6 years (predominantly preschool age), 7–9 years (early elementary school), 10–12 years (late elementary school), and 13–15 years (junior high school). Additional age groups were defined to represent later life stages, including high school students, young adults, university students, and early workforce entrants. All participants were drawn from the TMM BirThree Cohort Study and included both males and females across all age groups.

**Table 1.** Age, Sex, and Sample Collection Year of Participants Included in the CMV and EBV Seroprevalence Analyses

Characteristic	CMV Cohort, n (%)	EBV Cohort, n (%)
Age band (years), n (%)	2247	1647
4–6	200 (8.9)	200 (12.1)
7–9	200 (8.9)	200 (12.1)
10–12	220 (9.8)	220 (13.4)
13–15	206 (9.2)	206 (12.5)
16–18	220 (9.8)	220 (13.4)
19–21	55 (2.4)	55 (3.3)
22–24	146 (6.5)	146 (8.9)
25–27	200 (8.9)	200 (12.1)
28–30	200 (8.9)	200 (12.1)
31–33^a^	200 (8.9)	N/A
34–36^a^	200 (8.9)	N/A
37–40^a^	200 (8.9)	N/A
Sex, n (%)		
Male	1057 (47.0)	757 (46.0)
Female	1190 (53.0)	890 (54.0)
Collection year, n (%)		
2017	86 (3.8)	41 (2.5)
2018	703 (31.3)	491 (29.8)
2019	752 (33.5)	515 (31.3)
2020	309 (13.8)	227 (13.8)
2021	161 (7.2)	137 (8.3)
2022	80 (3.6)	80 (4.9)
2023	78 (3.5)	78 (4.7)
2024 (through April)	78 (3.5)	78 (4.7)

Abbreviations: CMV, cytomegalovirus; EBV, Epstein–Barr virus.

^a^EBV seroprevalence analyses were limited to participants aged ≤30 years by study design; CMV analyses included participants up to 40 years.

### Age-Stratified and Sex-Specific Seroprevalence of CMV and EBV

Figure 1 shows the age-stratified seroprevalences of EBV and CMV in males (Figure 1*A*) and females (Figure 1*B*) with 95% CIs. Because EBV seroprevalence approached 90% in males and 95% in females by approximately 30 years of age, EBV was analyzed in participants aged 4–30 years. In contrast, CMV was evaluated up to 40 years of age because seroprevalence remained approximately 81% in males and 68% in females at that age. For EBV, 757 males and 890 females were included (total, 1647), whereas for CMV, 1057 males and 1190 females were included (total, 2247). No marked differences in the overall seroprevalence patterns were observed between males and females for either virus. Epstein–Barr virus seroprevalence increased with age in both sexes, whereas CMV seroprevalence showed a gradual and less distinct age-related pattern.

**Figure 1:**
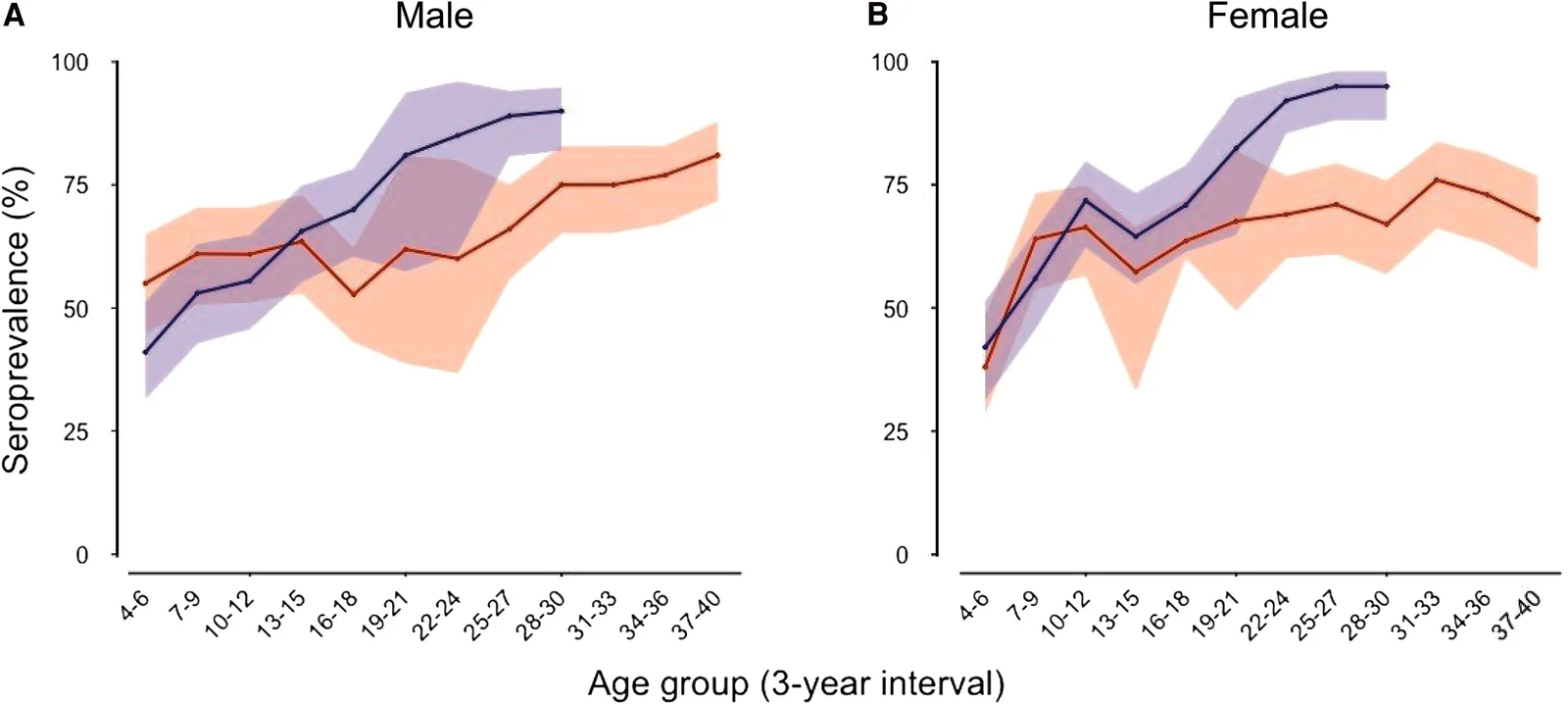
Age-specific seroprevalence of CMV and EBV by sex. Seroprevalence of CMV (red) and EBV (blue) by age and sex. Age was grouped into 3-year intervals. Points represent observed seroprevalence in each age group, and shaded areas indicate 95% confidence intervals. In both males (*A*) and females (*B*), CMV seroprevalence increased gradually with age, although the overall increase was modest. In contrast, EBV seroprevalence rose more sharply, reaching high levels (>90%) at a younger age. The analyses included individuals aged up to 40 years for the CMV and up to 30 years for the EBV.

CMV-IgG seroprevalence was already 55% in males and 38% in females at 4–6 years of age (Figure 1; Supplementary Table 1). In males, seroprevalence remained relatively stable across age groups, whereas in females, it increased with age, reaching 64% at 7–9 years and 68% at 37–40 years of age. In males, it remained relatively stable through adolescence before increasing to 81% at 37–40 years. Overall, CMV seroprevalence showed a slower and less distinct age-related increase than EBV seroprevalence.

EBV-IgG seroprevalence showed a steady increase with age, exceeding 70% in adolescents aged 16–18 years and reaching >90% by the late 20s. This rise was steeper than that of CMV and showed minimal sex differences (Figure 1*A* and *B*). Borderline antibody results were relatively common in younger age groups (4–18 years), but declined in adulthood, consistent with progressive seroconversion (Supplementary Table 2).

Sex-specific comparisons of seropositivity were performed within each age group. A significant difference between males and females was observed in the 4–6-year age group for CMV (*P* = .0231) and in the 10–12 year age group for EBV (*P* = .0169); however, no significant sex differences were found in the remaining age groups (Supplementary Table 3). Thus, no consistent pattern of sex-related differences in seropositivity was observed across age groups.

To assess whether reliance on VCA-IgG alone might have led to the misclassification of previously infected individuals as EBV-seronegative, additional EBV serological testing was performed in a subset of 88 samples. Among the samples, 50 were initially classified as VCA-IgG positive, 16 as borderline, and 22 as negative. Additional testing identified at least one positive EBV serological marker in 5 of the 16 VCA-IgG-borderline samples. In contrast, none of the 22 VCA-IgG-negative samples were positive for any of VCA-IgM, EA-IgG, or EBNA-IgG (Supplementary Table 4). These findings suggest that, within this retested subset, VCA-IgG negativity was not associated with evidence of previous EBV infection based on the additional serological markers.

Similar age- and sex-specific CMV and EBV seroprevalence patterns were observed when analyses were restricted to one randomly selected participant per family (Supplementary Table 5), indicating that these findings were not driven by household clustering.

### Co-positivity Patterns

Since EBV and CMV antibodies were measured in the same individuals, combinations of serostatuses for both viruses were further examined. The combined CMV and EBV antibody status showed age-dependent increases in dual seropositivity (CMV+/EBV+) and corresponding declines in the CMV−/EBV− group (Figure 2*A* and *D*). In both sexes, the proportion of seropositive individuals for both viruses gradually increased into adulthood, whereas the proportion of negative individuals for both viruses declined with age. The CMV+/EBV− subgroup (Figure 2*B*) also decreased with age, similar to the CMV−/EBV− subgroup. In contrast, the CMV−/EBV+ subgroup (Figure 2*C*) was observed across all age ranges and remained at a relatively constant proportion throughout life. These trends were similar in males and females, with minimal sex-specific differences. Notably, among EBV-seropositive individuals, CMV seropositivity remained incomplete in adulthood, with CMV positivity observed in 67% of males and 62% of females in the oldest age group, 28–30.

**Figure 2:**
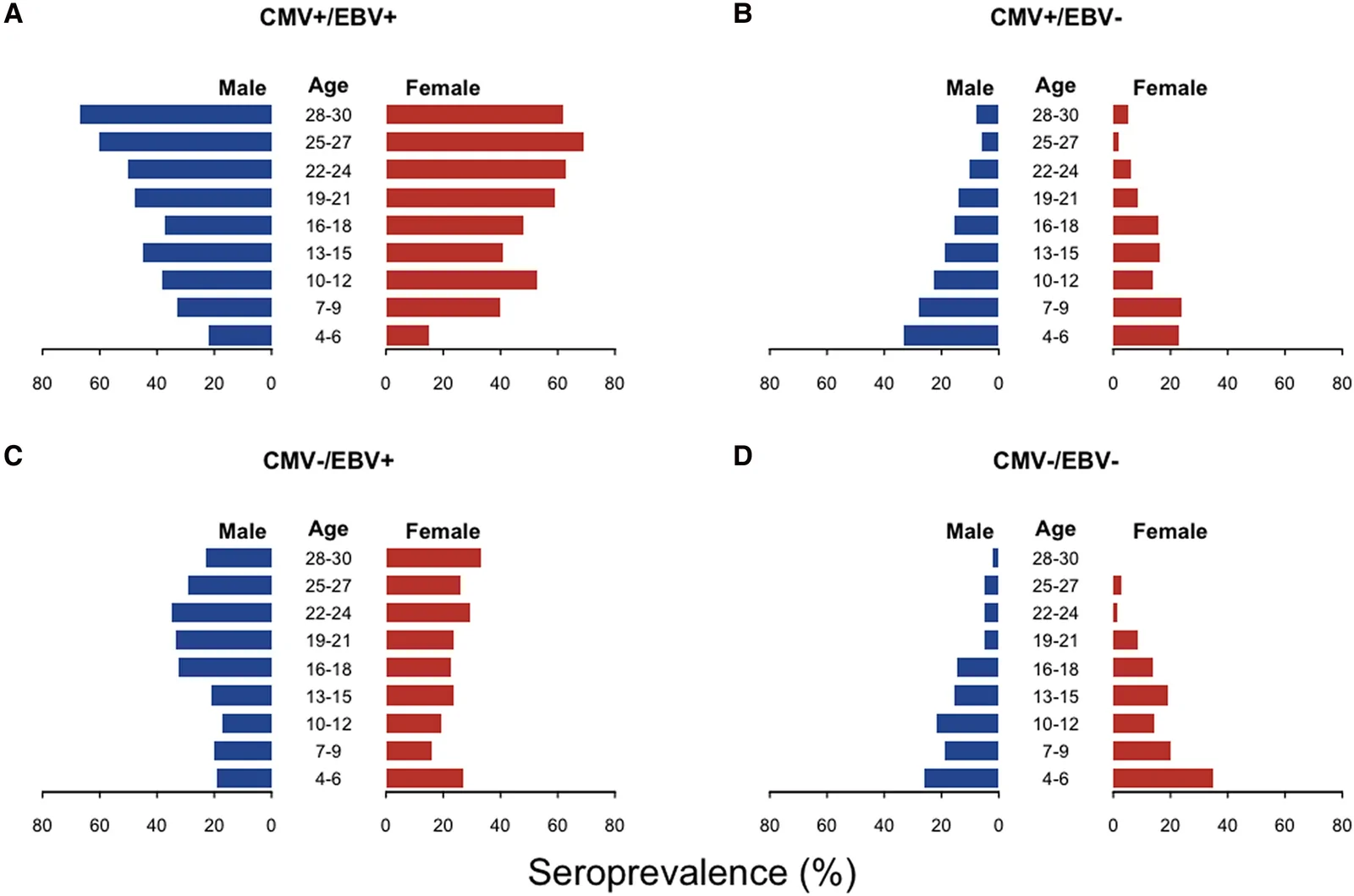
Age- and sex-specific seroprevalence by combined CMV/EBV serostatus. Age (3-year intervals)- and sex-specific seroprevalence patterns are shown for the 4 combined CMV/EBV serostatus groups: (*A*) CMV+/EBV+, (*B*) CMV+/EBV−, (*C*) CMV−/EBV+, and (D) CMV−/EBV−. Within each panel, proportions are displayed separately for males (left) and females (right) across age groups. Age groups are ordered from youngest (bottom) to oldest (top).

## DISCUSSION

In this population-based cohort of the TMM, we observed distinct age-related patterns of CMV and EBV seroprevalence among children, adolescents, and adults in Japan. Few population-based studies have evaluated CMV and EBV serostatus side by side in the same samples, from the same cohort. We observed that EBV-IgG seroprevalence increased steadily across age groups, exceeding 90% by the late 20s, whereas CMV-IgG seroprevalence plateaued at approximately 70%–80% in early adulthood. This divergence is notable despite their broadly similar transmission routes and suggests differences in the relative contribution or timing of transmission in Japan, with important public health implications.

The comparatively lower CMV seropositivity among women of reproductive age is of particular concern, as it implies that a substantial proportion of women remain susceptible to primary CMV infection during pregnancy, a key risk factor for congenital CMV infection [22]. A recent claims-based analysis in Japan reported a modest increase in the annual incidence of congenital CMV infections from 2011 to 2019 [23]. Approximately one-third of Japanese women in their 20s remain CMV-seronegative, underscoring the importance of evaluating maternal screening strategies [24], strengthening awareness of congenital CMV prevention, and developing targeted prevention programs.

For EBV, the observed pattern of delayed primary infection highlights the potential public health value of ongoing EBV vaccine development, particularly for the prevention of infectious mononucleosis and possibly for other EBV-associated conditions [25], such as multiple sclerosis and certain malignancies. Accordingly, understanding the age of acquisition may provide useful background information for the design of future preventive and post-exposure vaccination strategies.

The clinical significance of combined CMV and EBV serostatus may extend beyond the acquisition patterns of the 2 viruses. Several studies [26, 27] have reported that CMV seropositivity is inversely associated with the risk of multiple sclerosis, whereas EBV infection and strong antibody responses to EBV antigens are positively associated with the disease. Maple et al [28] have further suggested an interaction between CMV serostatus and EBV-specific immune responses. Although these observations speculate that CMV-induced immune modulation may influence EBV-associated immunopathology, the reported associations are observational, and neither a causal protective effect nor the underlying mechanism has been established. Further longitudinal studies will be needed to clarify whether combined CMV and EBV serostatus is associated with subsequent immune-mediated disease.

Our findings align with previous Japanese data showing high EBV seropositivity from late adolescence to adulthood [15, 16]. In contrast, CMV seroprevalence in this cohort was higher among children and adolescents but lower in adults than in earlier national estimates [14, 16], suggesting progressively later acquisition. Earlier surveys reported CMV seroprevalence exceeding 90% among adults, whereas our data plateaued around 70%–80% by age 40. Similar to other Japanese studies, CMV seroprevalence increased gradually with age, whereas EBV seroprevalence showed a sharp increase beginning in early childhood and rose rapidly by age 10. Together, these observations imply that while the overall EBV seroprevalence in Japan remains high and consistent with past reports, CMV exposure dynamics may be shifting alongside evolving demographic and behavioral factors.

Breastfeeding is considered an important potential route of postnatal CMV transmission and may contribute to CMV acquisition during infancy. We therefore explored whether participant characteristics, including infant feeding practices, differed between individuals who were seropositive by 5 years of age and those who remained seronegative after 20 years of age. However, the number of participants in these groups was small, limiting our ability to perform a meaningful comparison, and exploratory analyses using the available participant information did not identify any clear associations. Most participants had received mixed feeding, resulting in insufficient numbers in the exclusively breastfed and exclusively formula-fed groups for a reliable comparison. Moreover, information on breastfeeding duration and the relative proportions of breast milk and formula was unavailable. Thus, breastfeeding exposure could not be quantified, precluding a reliable assessment of its association with early CMV acquisition. Larger studies incorporating detailed socioeconomic, household, behavioral, environmental, and early-life exposure information are needed to identify factors associated with early or delayed acquisition of EBV and CMV infection in Japan.

The relatively delayed seroconversion of EBV in our cohort is consistent with recent seroepidemiological observations in Japan [16], suggesting that primary EBV infection may occur later in contemporary populations than in historical cohorts. While CMV is believed to be largely transmitted in early childhood, consistent with predominantly household-mediated transmission, EBV infection may be more strongly influenced by extra-household contact. However, the mechanisms underlying these distinct transmission patterns were not directly assessed in the present study. Notably, given that EBV seroprevalence approached 90% and CMV about 70% in the parental generation, household transmission may still play a substantial role in viral exposure.

The relatively high proportion of EBV-seronegative adults observed in this study may have resulted from the differences in assay performance. However, the diagnostic performance of EBV serological testing cannot be determined solely by the analytical platform, as it also depends on factors such as antigen composition, assay design, cutoff criteria, and the characteristics of the individual commercial kit. In the present study, a commercially available ELISA kit intended for clinical laboratory testing was used in accordance with the manufacturer's instructions. To further evaluate whether insufficient detection by the VCA-IgG assay could have contributed to the observed proportion of EBV-seronegative individuals, 88 available samples were additionally tested for VCA-IgM, EA-IgG, and EBNA-IgG. VCA-IgG showed the highest positivity rate among the 4 serological markers, followed by EBNA-IgG, whereas EA-IgG was detected only rarely. Moreover, most samples that were positive/borderline for EBNA-IgG or EA-IgG were also positive for VCA-IgG. These findings suggest that the relatively high proportion of EBV-seronegative adults was unlikely to be explained primarily by insufficient detection using the VCA-IgG ELISA assay. Nevertheless, because a direct comparison with a chemiluminescent immunoassay was not performed, possible differences in analytical performance between assay platforms cannot be excluded.

Globally, EBV seroprevalence remains high, although several studies have reported that the age of primary infection has shifted away from early childhood in Europe, North America, and parts of East Asia [29–31]. This shift may underlie the increasing frequency of infectious mononucleosis [17, 32] and, indirectly, the rising prevalence of multiple sclerosis observed in Japan [9]. In contrast, a recent systematic review demonstrated markedly lower CMV seroprevalence in high-income countries than in low- and middle-income countries, particularly among women of reproductive age, with seropositivity gradually increasing with age [33]. These global data support the hypothesis that delayed CMV acquisition is accompanied by socioeconomic development, smaller household size, and reduced exposure to young children.

The contrasting age-related trajectories of CMV and EBV may reflect differences in biological persistence, transmission efficiency, and host–virus interactions, despite partially overlapping transmission routes, such as saliva and close interpersonal contact. Differences in viral shedding kinetics, infectious doses, and the duration of humoral immunity may contribute [34, 35]. Sociodemographic factors, such as household composition, daycare exposure, and number of siblings, likely influence CMV exposure more than EBV. These factors may help explain why some individuals remain seronegative for both viruses into adolescence and adulthood.

Although an isolated significant difference in seropositivity between males and females was observed in one age group for each virus, no consistent sex-related pattern was evident across the age groups. Therefore, these findings do not provide clear evidence that sex was a major determinant of CMV or EBV seropositivity in this population. Because multiple comparisons were performed across age groups, the isolated significant results should be interpreted cautiously and may have occurred by chance. Differences in exposure patterns, household contact, childcare practices, or other behavioral and demographic factors could potentially contribute to sex-specific seropositivity; however, such information was not available in the present study, and the observed isolated differences cannot be attributed to a specific mechanism.

A notable finding of this study was the persistence of individuals seronegative for both EBV and CMV during adolescence and early adulthood. Even in the 16–18-year age group, dual seronegativity was observed in 14.5% of males and 13.6% of females (Figure 2*D*). This is clinically relevant because primary infection during the reproductive years, particularly with CMV, may be associated with adverse pregnancy outcomes, including congenital infections and fetal complications. Dual seronegativity remained detectable in the twenties, affecting approximately 5% of males and up to 3% of females, indicating that a subset of young adults remained at risk for primary infection. However, dual seronegativity became uncommon in later adulthood, suggesting progressive acquisition of at least one infection over time. Although EBV and CMV are both human herpes viruses with partially overlapping transmission opportunities, simultaneous evaluation of both antibodies in the same participants revealed patterns that may not be readily captured in studies assessing each virus separately or in different cohorts and represents a distinctive strength of this study.

The samples collected between 2017 and 2024 were analyzed as a single dataset (Table 1). Although COVID-19-related infection-prevention measures substantially altered the epidemiology of several pediatric infections in Japan, an exploratory comparison of samples collected during 2017–2019 and from 2020 onward was limited by marked differences in sample size and age distribution between the periods [36]. Therefore, the potential influence of the COVID-19 pandemic on EBV and CMV acquisition could not be evaluated reliably. Longitudinal or adequately age-matched studies are needed to address this issue.

This study benefits from its large, population-based design, use of standardized assays, and contemporary data collected across multiple age strata. However, several limitations warrant consideration. The participants were not randomly selected from the entire Japanese population, and the results may not be generalizable beyond the Tohoku region. Because the cohort was family structured, including siblings, parents, and grandparents, observations within families may not be fully independent. Although this design provided broad age coverage, it also introduced potential clustering effects; however, similar age-specific seroprevalence patterns were observed when the analyses were restricted to one randomly selected participant per family. Residual confounding factors related to socioeconomic, behavioral, or environmental variables cannot be excluded. Only IgG antibody data were available, which precluded the assessment of functional or cellular immunity.

## CONCLUSION

In summary, this study provides updated evidence of CMV and EBV seroprevalence in Japan, revealing divergent infection dynamics despite shared transmission pathways. The early plateau in CMV seroprevalence and delayed EBV acquisition indicate changing exposure patterns in younger generations. Continued surveillance and integrated epidemiologic and virologic studies are needed to clarify these trends and guide vaccine strategy and maternal-child health prevention efforts.

## Supplementary Material

ofag678_Supplementary_Data

## Supplementary Data

Supplementary materials are available at *Open Forum Infectious Diseases* online. Consisting of data provided by the authors to benefit the reader, the posted materials are not copyedited and are the sole responsibility of the authors, so questions or comments should be addressed to the corresponding author.
