# Supplementary material for: Age-Stratified Seroprevalence of Cytomegalovirus and Epstein–Barr Virus in a Population-Based Cohort in Japan, 2017–2024
Source: Open Forum Infect Dis. 2026 Nov 4;13(11):ofag678. doi: 10.1093/ofid/ofag678 (PMC13755887; doi:10.1093/ofid/ofag678)
Supplement: Supplementary file 1 [file ofag678_supplementary_data.docx]

**Supplementary data**

**Table S1:** **Age-specific cytomegalovirus IgG seroprevalence by sex among 4–18-year-old participants in the TMM BirThree Cohort Study**

| **Age group (years)** | **Sex** | **Seronegative**  **n (%)** | **Borderline**  **n (%)** | **Seropositive**  **n (%)** | **Total**  **n** |
| --- | --- | --- | --- | --- | --- |
| 4–6 | Male | 44 (44.0) | 1 (1.0) | 55 (55.0) | 100 |
|  | Female | 62 (62.0) | 0 (0.0) | 38 (38.0) | 100 |
| 7–9 | Male | 38 (38.0) | 1 (1.0) | 61 (61.0) | 100 |
|  | Female | 35 (35.0) | 1 (1.0) | 64 (64.0) | 100 |
| 10–12 | Male | 41 (37.3) | 2 (1.8) | 67 (60.9) | 110 |
|  | Female | 34 (30.9) | 3 (2.7) | 73 (66.4) | 110 |
| 13–15 | Male | 33 (34.4) | 2 (2.1) | 61 (63.5) | 96 |
|  | Female | 45 (40.9) | 2 (1.8) | 63 (57.3) | 110 |
| 16–18 | Male | 51 (46.4) | 1 (0.9) | 58 (52.7) | 110 |
|  | Female | 40 (36.4) | 0 (0.0) | 70 (63.6) | 110 |

IgG antibodies were measured using a commercial enzyme-linked immunosorbent assay kit (Denka Co., Ltd., Tokyo, Japan). Columns show the number and percentage of participants classified as seronegative, seropositive, or borderline. Data for adults (≥19 years) are not shown.

**Table S2. Age-specific Epstein–Barr virus IgG seroprevalence by sex among 4–18-year-old participants in the TMM BirThree Cohort Study**

| **Age group (years)** | **Sex** | **Seronegative**  **n (%)** | **Borderline**  **n (%)** | **Seropositive**  **n (%)** | **Total n** |
| --- | --- | --- | --- | --- | --- |
| 4–6 | Male | 41 (41.0) | 18 (18.0) | 41 (41.0) | 100 |
|  | Female | 45 (45.0) | 13 (13.0) | 42 (42.0) | 100 |
| 7–9 | Male | 32 (32.0) | 15 (15.0) | 53 (53.0) | 100 |
|  | Female | 36 (36.0) | 8 (8.0) | 56 (56.0) | 100 |
| 10–12 | Male | 36 (32.7) | 13 (11.8) | 61 (55.5) | 110 |
|  | Female | 21 (19.1) | 10 (9.1) | 79 (71.8) | 110 |
| 13–15 | Male | 26 (27.1) | 7 (7.3) | 63 (65.6) | 96 |
|  | Female | 28 (25.5) | 11 (10.0) | 71 (64.5) | 110 |
| 16–18 | Male | 27 (24.5) | 6 (5.5) | 77 (70.0) | 110 |
|  | Female | 23 (20.9) | 9 (8.2) | 78 (70.9) | 110 |

IgG antibodies against the Epstein–Barr virus capsid antigen were measured using commercial enzyme-linked immunosorbent assay kits (Denka Co., Ltd., Tokyo, Japan). Columns show the number and percentage of participants classified as seronegative, seropositive, or borderline. Data for adults (≥19 years) are not shown.

**Table S3. Comparison of CMV and EBV seropositivity between males and females according to age group**

| *P*-value | | |
| --- | --- | --- |
| Age group (years) | CMV | EBV |
| 4–6 | **0.0231** | 1 |
| 7–9 | 0.77 | 0.777 |
| 10–12 | 0.484 | **0.0169** |
| 13–15 | 0.394 | 0.885 |
| 16–18 | 0.133 | 1 |
| 19–21 | 0.773 | 1 |
| 22–24 | 0.446 | 0.389 |
| 25–27 | 0.543 | 0.191 |
| 28–30 | 0.275 | 0.283 |
| 31–33 | 1 | NA |
| 34–36 | 0.624 | NA |
| 37–40 | 0.0509 | NA |

Data are presented as the number of seropositive participants/total number tested (%). Borderline/equivocal antibody results were classified as seronegative for the purpose of calculating seropositivity rates and performing statistical analyses. Differences between males and females within each age group were evaluated using Fisher’s exact test. All tests were two-sided, and *P-values* < 0.05 were considered statistically significant and are shown in bold. *P-values* were not adjusted for multiple comparisons.

CMV, cytomegalovirus; EBV, Epstein–Barr virus; NA, not available.

**Table S4. EBV antibody profiles according to initial VCA-IgG results in 88 retested samples**

|  |  | Positive for | | | |
| --- | --- | --- | --- | --- | --- |
| VCA-IgG | n | VCA-IgM | EA-IgG | EBNA-IgG | Any additional marker |
| Positive | 50 | 6 | 0 | 39 | 39 |
| Borderline | 16 | 1 | 0 | 4 | 5 |
| Negative | 22 | 0 | 0 | 0 | 0 |
| Total | 88 | 7 | 0 | 43 | 44 |

Testing positive for any additional marker was defined as positivity for at least one of VCA-IgM, EA-IgG, or EBNA-IgG. VCA-IgG results were classified as positive, borderline, or negative according to the manufacturer-defined cutoff values.

VCA, viral capsid antigen; EA, early antigen; EBNA, Epstein–Barr nuclear antigen.

**Table S5. Age- and sex-stratified cytomegalovirus and Epstein–Barr virus IgG seroprevalence among one randomly selected participant per family**

| **Age group (years)** | **CMV IgG positive** | | **EBV IgG positive** | |  |
| --- | --- | --- | --- | --- | --- |
|  |  |  |  |  |  |
|  | **Male** | **Female** | **Male** | **Female** |  |
|  | **n/N (%) [95 % CI]** | **n/N (%) [95 % CI]** | **n/N (%) [95 % CI]** | **n/N (%) [95 % CI]** |  |
| **4–6** | 54/93 (58.1) [47.4-68.1] | 33/90 (36.7) [26.9-47.5] | 37/93 (39.8) [29.9-50.5] | 37/90 (41.4) [31.0-52.0] |  |
| **7–9** | 40/66 (60.6) [47.8-72.2] | 46/69 (66.7) [54.2-77.3] | 31/66 (47.0) [34.7-59.6] | 37/69 (53.6) [41.3-65.6] |  |
| **10–12** | 47/77 (61.0) [49.2-71.7] | 42/70 (60.0) [47.6-71.3] | 41/77 (53.2) [41.6-64.6] | 46/70 (65.7) [53.3-76.4] |  |
| **13–15** | 35/57 (61.4) [47.6-73.7] | 33/57 (57.9) [44.1-70.6] | 35/57 (61.4) [47.6-73.7] | 38/57 (66.7) [52.8-78.3] |  |
| **16–18** | 33/58 (56.9) [43.3-69.6] | 41/67 (61.2) [48.5-72.6] | 35/58 (60.3) [46.6-72.7] | 49/67 (73.1) [60.7-82.9] |  |
| **19–21** | 4/7 (57.1) [20.2-88.2] | 7/12 (58.3) [28.6-83.5] | 5/7 (71.4) [30.3-94.9] | 9/12 (75.0) [42.8-93.3] |  |
| **22–24** | 5/7 (71.4) [30.3-94.9] | 62/91 (68.1) [57.4-77.3] | 5/7 (71.4) [30.3-94.9] | 85/91 (93.4) [85.7-97.3] |  |
| **25–27** | 46/64 (71.9) [59.0-82.1] | 54/73 (74.0) [62.2-83.2] | 56/64 (87.5) [76.3-94.1] | 70/73 (95.9) [87.7-98.9] |  |
| **28–30** | 64/87 (73.6) [62.8-82.2] | 55/79 (69.6) [58.1-79.2] | 78/87 (89.7) [80.8-94.9] | 75/79 (94.9) [86.9-98.4] |  |
| **31–33** | 60/82 (73.2) [62.1-82.1] | 67/87 (77.0) [66.5-85.1] | N/A | N/A |  |
| **34–36** | 69/89 (77.5) [67.2-85.4] | 53/75 (70.7) [58.9-80.3] | N/A | N/A |  |
| **37–40** | 71/88 (80.7) [70.6-88.0] | 55/79 (69.6) [58.1-79.2] | N/A | N/A |  |

CI, confidence interval; 95% CI shown where applicable. CMV, cytomegalovirus; EBV, Epstein–Barr virus; NA, not available.
